# Identification of factors associated with hospitalization in an outpatient population with mental health conditions: a case–control study

**DOI:** 10.3389/fpsyt.2024.1341160

**Published:** 2024-04-18

**Authors:** Matthieu Lebrat, Rachel Megard, Cédric Dananché, Luc Zimmer, Julien Plasse, Nicolas Franck

**Affiliations:** ^1^ Pôle Centre Rive Gauche, CH Le Vinatier, Bron, France; ^2^ Université Claude Bernard Lyon 1, Villeurbanne, France; ^3^ Pôle Santé Publique, Hospices Civils de Lyon, Lyon, France; ^4^ UMR 5992 CNRS, U1028 INSERM, Centre de Recherche en Neurosciences de Lyon, Bron, France; ^5^ Hospices Civils de Lyon, Lyon, France; ^6^ UMR 5229 CNRS, Centre Ressource de Réhabilitation psychosociale, Le Vinatier, Bron, France

**Keywords:** mental health system, outpatient clinic, deinstitutionalization, epidemiology, public health

## Abstract

**Introduction:**

Addressing relevant determinants for preserved person-centered rehabilitation in mental health is still a major challenge. Little research focuses on factors associated with psychiatric hospitalization in exclusive outpatient settings. Some variables have been identified, but evidence across studies is inconsistent. This study aimed to identify and confirm factors associated with hospitalization in a specific outpatient population.

**Methods:**

A retrospective monocentric case-control study with 617 adult outpatients (216 cases and 401 controls) from a French community-based care facility was conducted. Participants had an index outpatient consultation between June 2021 and February 2023. All cases, who were patients with a psychiatric hospitalization from the day after the index outpatient consultation and up to 1 year later, have been included. Controls have been randomly selected from the same facility and did not experience a psychiatric hospitalization in the 12 months following the index outpatient consultation. Data collection was performed from electronic medical records. Sociodemographic, psychiatric diagnosis, historical issues, lifestyle, and follow-up-related variables were collected retrospectively. Uni- and bivariate analyses were performed, followed by a multivariable logistic regression.

**Results:**

Visit to a psychiatric emergency within a year (adjusted odds ratio (aOR): 13.02, 95% confidence interval (CI): 7.32–23.97), drug treatment discontinuation within a year (aOR: 6.43, 95% CI: 3.52–12.03), history of mental healthcare without consent (aOR: 5.48, 95% CI: 3.10–10.06), medical follow-up discontinuation within a year (aOR: 3.17, 95% CI: 1.70–5.95), history of attempted suicide (aOR: 2.50, 95% CI: 1.48–4.30) and unskilled job (aOR: 0.26, 95% CI: 0.10–0.65) are the independent variables found associated with hospitalization for followed up outpatients.

**Conclusions:**

Public health policies and tools at the local and national levels should be adapted to target the identified individual determinants in order to prevent outpatients from being hospitalized.

## 1 Introduction

The deinstitutionalization process in psychiatry began in the late twentieth century. This shift, especially seen in high-income countries, consists of a decrease in specialized psychiatric hospital beds for an increase of patients with a mental health condition, followed up in general medical hospitals, community-based care, and various outpatient settings (1). Between the mid-twentieth century and the 1990s, the number of psychiatric beds dropped to more than 80% in most western regions around the world (1).

However, the transition from an inpatient setting paradigm to an outpatient one needs to be carefully organized, with the necessary and appropriate structures and funding. Indeed, patients who suffer from a mental health disease need a deep consideration of the multifaceted world in which they live, to integrate and adapt their rehabilitation process for the outside world. The strengthening of community services has been heterogenous around the world (1). This deinstitutionalization failed, for example, in many places in the USA, leading to an increase in homelessness and crime among people with psychiatric diseases in the 1990s (2). More recently, there are still concerns about the good transitioning process that have been raised in central and eastern Europe, with a large body of evidence showing failures in deinstitutionalization and reinstitutionalization outcomes. Some of the causes found are lack of personal assistance, development and adaptation of social housing, and cuts to social support (3). The limited scaling up of community-based and primary care mental health services has also been identified as a failure factor of deinstitutionalization, along with fundamental concerns with the model. A deeper work on addressing social determinants is indeed also evoked, which are known to be fundamental structural drivers of mental illness (1). A relatively recent dramatic event that has to be remembered regarding the deinstitutionalization failure has been the “Life Esidimeni scandal” in 2016 in South Africa. Qualified as a humanitarian crisis, this event caused the deaths of a thousand psychiatric patients (94 according to an official report issued in 2017 (4)) following their transfer from an inpatient setting to multiple outpatient settings without the appropriate care and follow-up required. Indeed, the cut in this 2,000-bed facility budget led to patients’ discharge regardless of individual autonomy and psychosocial disability into inadequately resourced nongovernmental facilities (5).

Deinstitutionalization requires strong, continuous efforts and should always stay person-centered. In this approach, the multidisciplinary team caring for the patient must bear in mind the individual factors that can predict the maintained recovery of the patient in the outpatient setting (6). Few settings succeed yet to address all structural determinants, even in high-income countries (1). Indeed, the *Lancet* Commission on Global Mental Health and Sustainable Development reminded us that regarding mental health, all countries are “developing” due to the relative underfunding of mental health services in relation to the burden of the condition (7). Ways to achieve success with deinstitutionalization may involve legislation with a mandate to establish community-based services (like in Italy (8)) and to adapt them to a local context. Improvements will probably require a multitude of paradigm shifts within these structures, considering factors enabling their enhancement. If no adequate care is provided during deinstitutionalization or after it, patients may relapse after being discharged from the hospital and consequently readmitted. Many studies therefore considered readmission rate to be an indicator for intervention studies (1) and to identify protective and risk factors of relapses (9) (10, 11).

A rich scientific literature is available on the study of risk factors of hospitalization in patients suffering from mental health pathologies. Nonexhaustively, for depression (12), the type of illness diagnosis, psychiatric comorbidity, treatment-related factors, and sociodemographic factors were associated with hospitalization. For bipolar disorders (13), characteristics of the index hospitalization (transfer, discharge disposition, length of stay), all-cause acute health service utilization in the year prior to it, and comorbidity were identified. For schizophrenia (14, 15), recent medical follow-up discontinuation, medication nonadherence, life events, comorbidity, sex, age, and medication type were variables associated with hospitalization. Finally, for other psychiatric conditions (16) (9, 10) (17) (11) (18), factors associated with hospitalization were shown to be recent medical follow-up discontinuation, multiple psychiatric hospitalization history, history of mental healthcare without consent, social isolation, socioeconomic status, violence history, psychiatric diagnosis, and patient’s satisfaction with treatment. A suicide attempt was found to be a risk factor for hospitalization in some studies and a protective factor at 1 year in others.

Nonetheless, the studies cited above only evaluate risk factors for readmission, i.e., for patients that are originally coming from an inpatient hospital setting. Literature focusing on an exclusive outpatient setting is scarce (19, 20). It confirmed some previously identified risk factors in studies with an inpatient setting, such as alcohol/substance use, family history of mental health disease, and marital status, but have also diverging results for negative attitude/poor compliance with medication, identified by Antonio Ciudad et al. (20) as lowering the hazard of relapse during outpatient follow-up.

A systematic review of the literature carried out by Donisi et al. (11) additionally underlined some inhomogeneous results for identified risk factors associated with readmissions regarding sociodemographic variables, and a literature weakness for social support, considered only in a few papers. Furthermore, the authors emphasized that some factors were only identified in uni- or bivariate analyses and not in multiple regression.

More people are followed up in outpatient settings, and the minimal use of hospitalization remains a challenge in mental health. This study is of interest to mental health professionals and policymakers because more data on factors associated with hospitalization in followed up outpatients could help tailor appropriate follow-up care and adapt existing tools to reduce the need for hospitalization. Our study, therefore, aimed to identify and confirm risk factors of hospitalization in a specific outpatient population.

## 2 Methods

### 2.1 Study design

We conducted an observational, retrospective, monocentric case-control study based on hospitalization in one of the largest university-affiliated public psychiatric hospitals in France, with around 500 beds and 26,500 patients followed up on an outpatient basis, the Centre Hospitalier le Vinatier (CHV) in Bron. The CHV has several community-based care facilities called “Centre Médico-Psychologique” or “CMP”, providing medical–psychological and social consultations to anyone experiencing psychological difficulties. The present study was made in one of them. We reported this case-control study according to Strengthening the Reporting of Observational Studies in Epidemiology (STROBE). For details, see **Supplementary File 1**.

### 2.2 Sample

This retrospective study investigated the data from patients followed up in an outpatient setting from June 2021 to February 2023. This study period has been chosen in order not to have repercussions of the health restrictions due to the COVID-19 pandemic on our variables. The studied sample comes from the *Centre Médico-Psychologique Centre Rive Gauche* facility, administratively attached to the CHV but which has an independent operation for outpatients requiring mental healthcare in a defined geographic area (third, sixth, and eighth districts of Lyon).

In this facility, participants were eligible if they were aged 18 or older and had at least one outpatient psychiatric medical consultation between June 2021 and February 2023 (defined as the index consultation).

The sample size for this study was determined considering an odds ratio of 1.5 to 3 clinically meaningful based on previous literature. With a significance level of 0.05, a type I error of 0.025, and a power of 0.9, the required sample size was calculated using R and its Epicalc package 2.9.0.1. An estimate was then made with the lowest and highest expected frequencies for the studied variables. An ideal sample size was calculated and ranged between 807 and 423, with an approximate 1:2 case/control ratio.

### 2.3 Outcome

The studied outcome was full psychiatric hospitalization from the day after the index outpatient consultation and up to 1 year later. Full psychiatric hospitalization was defined in this study as more than 24 h of hospitalization in a psychiatric hospital. Thus, participants who had this outcome of interest were referred to as cases, whereas others who did not have the outcome of interest were referred to as controls.

### 2.4 Selection of cases and controls

Cases were patients who had a full psychiatric hospitalization from the day after the index outpatient consultation and up to 1 year later.

Controls were patients who did not experience full psychiatric hospitalization in the 12 months following the index outpatient consultation (therefore, controls have an index outpatient consultation before February 2022 to have at least a 1-year psychiatric hospitalization-free period).

All cases in the sample responding to the case definition were included (*n* = 216).

Controls (*n* = 401) were then randomly selected from the sample list of patients who met the definition of controls in order to approximately respect a 1:2 case/control ratio and the sample size determination. The random selection was performed with simple random sampling using computer-generated random numbers to ensure an unbiased selection process.

All the detailed characteristics of cases and controls can be found in **Table 1**.

**Table 1:** Descriptive analysis.

Variable	Cases, full psychiatric hospitalization from the day after the index outpatient consultation and up to 1 year later (*n* = 216)	Controls, without full psychiatric hospitalization in the 12 months following the index outpatient consultation (*n* = 401)	*p*-value (for *N*)
*Demographic variables* N (%)†			
Age in years (*mean (sd)*)	42.7 (13.9)	45.1 (13.0)	0.032^*^
Gender			
Female	75 (34.7)	176 (43.9)	0.027^*^
Male	141 (65.3)	225 (56.1)	
Birth in France	156 (72.2) [/215]	303 (75.6) [/396]	0.280
*Employment status*			
Unemployed	164 (75.9)	252 (62.8)	< 0.001^**^
Unskilled worker	11 (5.1)	72 (18.0)	< 0.001^**^
Homeless	29 (13.4)	10 (2.5)	<0.001^**^
Partner	53 (24.5) [/215]	110 (27.4) [/400]	0.445
*Main diagnosis*			
Schizophrenia or other primary psychotic disorders	144 (66.7)	206 (51.4)	< 0.001^**^
Depressive disorders	20 (9.3)	62 (15.5)	0.030^*^
Bipolar or related disorders	34 (15.7)	45 (11.2)	0.109
Anxiety or fear-related disorders	4 (1.9)	32 (8.0)	0.002^*^
Neurodevelopmental disorders	2 (1.0)	22 (5.5)	0.005^*^
Other psychiatric diagnosis	12 (5.0)	34 (8.5)	0.358
Psychiatric comorbidity	59 (27.3)	119 (30.0)	0.819
*Historical issues and lifestyle*			
Traumatic history	134 (62.0) [/203]	226 (56.4) [/376]	0.162
History of mental healthcare without consent	181 (83.8) [/215]	187 (46.6) [/398]	< 0.001^**^
Multiple psychiatric hospitalization history (>5)	143 (66.2)	130 (32.4)	< 0.001^**^
Alcohol abuse^y^	52 (24.1)	40 (10.0) [/400]	< 0.001^**^
Illicit drug abuse^y^	62 (28.7) [/215]	52 (13.0) [/400]	< 0.001^**^
Family history of mental health disease	112 (51.9) [/193]	170 (42.4) [/369]	0.007^*^
History of attempted suicide	116 (53.7) [/210]	140 (34.9) [/390]	< 0.001^**^
Drug side effects^y^	55 (25.5)	96 (23.9)	0.675
*Follow-up-related variables*			
Visit to psychiatric emergency^y^	130 (60.2)	31 (7.7)	< 0.001^**^
Drug treatment discontinuation^y^	126 (58.3) [/214]	50 (12.5)	< 0.001^**^
Medical follow-up discontinuation^y^	106 (49.1)	56 (14.0)	< 0.001^**^
Additional support^y^	182 (84.3)	376 (93.8)	< 0.001^**^
Time since first admission to the psychiatric hospital in years (*mean (sd)*)	11.5 (7.37)	11.6 (7.31)	0.746

Sociodemographic, clinical, personal history, and follow-up characteristics of cases and controls from the outpatient public community-based care (Centre Médico-Psychologique Centre Rive Gauche, for outpatients of the third, sixth, and eighth districts of Lyon, France, requiring mental health follow-up), N = 617.

N (%), number (or mean when stated) of cases and controls; SD, standard deviation.

^*^p < 0.05; ^**^p < 0.001—determined using Student’s t-test for quantitative variables and Chi-square (χ^2^) test for qualitative variables.

+ When there are missing data, the total number of nonmissing observations for each variable is indicated in square brackets.

y Occurrence within the year (12 months prior to hospitalization for cases or following index consultation for controls).

### 2.5 Variables

The following exposure or potential confounder variables were collected retrospectively from patients’ medical records (collected in a binary yes/no format for qualitative variables):

- Sociodemographic variables: age (in years, quantitative variable), gender, birth in France, unemployed (including patients on sick leave but not retired patients), unskilled worker (i.e., job accessible without special qualifications, only job category collected), homeless, partner of life (in a relationship).
- Main psychiatric diagnosis (1 only), according to the ICD-11: depressive disorders, schizophrenia or other primary psychotic disorders, bipolar or related disorders, anxiety or fear-related disorders, neurodevelopmental disorders, another psychiatric diagnosis (other diagnosis belonging to the ICD-11 category 6: mental, behavioral, or neurodevelopmental disorders).
- Psychiatric comorbidity: the presence of a psychiatric comorbidity (in addition to the main diagnosis, the presence of another psychiatric disorder falling under category 6 of the ICD-11).
- Historical issues and lifestyle: traumatic history (exhaustively: rape and/or sexual assault and/or loss of first-degree relative before the patient’s age of 18 and/or torture and/or major physical assault and/or loss of a child by suicide and/or violent death of a first-degree relative in front of the patient and/or patient placed in foster care during childhood, and/or direct witness to a homicide), history of mental healthcare without consent (medical treatment undertaken without the consent of the patient being treated, as permitted by law), multiple psychiatric hospitalization history (> 5 full psychiatric hospitalization), alcohol abuse within the year (diagnosed by the psychiatrist as pathologic, and corresponding to the ICD-11 codes 6C40.0, 6C40.1, 6C40.20, 6C40.21, and 6C40.3), illicit drug abuse within the year (regular consumption of an illicit substance greater than 1/week), family history of mental health disease (known psychiatric disorder within the patient’s biological family), history of attempted suicide, and drug side effect reported within the year (presence of a side effect documented on the patient’s medical record).
- Follow-up-related variables: visit to psychiatric emergency within the year (excluding the one that led to full psychiatric hospitalization of the case definition), drug treatment discontinuation within the year (discontinuation by the patient, without medical agreement, of a psychiatric background treatment regimen over a period of more than 1 week), medical follow-up discontinuation within the year, additional support within the year (follow-up by a psychiatrist at least twice a year and/or regular follow-up by a medical mobile team (> 1/trimester) and/or included in a psychoeducation care program with a total hourly volume > 15 h/year), and time since first admission to the psychiatric hospital in outpatient or inpatient setting (in years, quantitative variable).

The term “within the year” refers to the variable being present 12 months prior to hospitalization for cases or 12 months following the index consultation for controls.

These variables were chosen because they have already been identified in the literature as factors associated with psychiatric hospitalization or suggested to be potential risk factors or confounders.

We hypothesized that all variables might be potential confounders and were indiscriminately tested to include them in the regression model (see Section 2.6) and to control for potential confounders.

### 2.6 Analysis

Statistical analysis was conducted using R software version 4.2.1 (23 June 2022) (R Core Team, 2022). Collected variables in case and control groups have been compared using a bivariate analysis (**Table 1**). For quantitative variables, the Student’s *t*-test was used. For qualitative variables (dichotomous variables collected in a yes/no format), a Chi-square (*χ* ^2^) test was performed.

Multivariable logistic regression was used to study the relationship between the outcome and the assessed covariables (listed in Section 2.2) with adjusted odds ratios (aORs) and 95% confidence intervals (CIs). In the analysis and to interpret its results, control group variables were considered baseline/reference category and case variables were compared to them. Based on the significant factors identified in the univariate analysis, variables were added to the model when *p* < 0.10. The model was built using a forward, stepwise selection procedure. It involves iteratively adding variables to the model one at a time, based on their individual contribution to improving the model’s fit. The fitness of the models was compared with a likelihood-ratio test. The choice was made to work on a subset of patients without missing data (complete case analysis). Interactions between variables included in the model were tested. They were considered when they appeared significant (*p*-value < 0.01 to avoid multiple testing problems) and had an interpretable clinical meaning. The multiple logistic regression model was adjusted for all the risk factor variables included in the full model (**Table 2**). The data normality of residuals for this multiple logistic regression was assessed by the Shapiro–Wilk normality test.

**Table 2:** Univariate analysis and results of a multiple logistic regression model predicting psychiatric hospital admission of outpatients (on a no missing values dataset, *n* = 521).

Variables	Univariate analysis		Multiple logistic regression model	
	Comparison of hospitalized and controls	*p*-value	Comparison of hospitalized and controls	*p*-value
	OR^a^ (95% CI)		aOR^a^ (95% CI)	
*Demographic variables*				
Age (in years)	0.99 (0.972–1.00)	0.044		
Sex (male)	1.64 (1.12–2.39)	0.009^*^		
Birth in France	0.91 (.59–1.39)	0.668		
*Employment status*				
Unemployed	1.98 (1.32–3.02)	< 0.001^**^		
Unskilled worker	0.26 (0.12–0.50)	< 0.001^**^	0.26 (0.10–0.65)	0.006^*^
Homeless	5.74 (2.47–14.94)	< 0.001^**^		
Partner	0.84 (0.56–1.26)	0.423		
*Main diagnosis*				
Schizophrenia or other primary psychotic disorders	1.92 (1.32–2.81)	< 0.001^**^		
Depressive disorders	0.54 (0.29–0.94)	0.030^*^		
Bipolar or related disorders	1.45 (0.86–2.40)	0.153		
Anxiety or fear-related disorders	0.18 (0.04–0.53)	< 0.001^**^		
Neurodevelopmental disorders	0.19 (0.03–0.67)	0.007^*^		
Other psychiatric diagnosis	0.74 (0.31–1.58)	0.450		
Psychiatric comorbidity	0.89 (0.59–1.33)	0.597		
*Historical issues and lifestyle*				
Traumatic history	1.22 (0.84–1.78)	0.280		
History of mental healthcare without consent	5.95 (3.87–9.40)	< 0.001^**^	5.48 (3.10–10.06)	< 0.001^**^
Multiple psychiatric hospitalization history (>5)	4.12 (2.82–6.08)	< 0.001^**^		
Alcohol abuse ^y^	2.80 (1.72–4.59)	< 0.001^**^		
Illicit drug abuse ^y^	2.95 (1.89–4.65)	< 0.001^**^		
Family history of mental health disease	1.66 (1.15–2.41)	0.006^*^		
History of attempted suicide	2.20 (1.52–3.19)	< 0.001^**^	2.50 (1.48–4.30)	< 0.001^**^
Drug side effects^y^	1.22 (0.80–1.83)	0.336		
*Follow-up-related variables*				
Visit to psychiatric emergency^y^	16.07 (9.95–26.73)	< 0.001^**^	13.02 (7.32–23.97)	< 0.001^**^
Drug treatment discontinuation^y^	8.16 (5.32–12.71)	< 0.001^**^	6.43 (3.52–12.03)	< 0.001^**^
Medical follow-up discontinuation^y^	4.89 (3.23–7.47)	< 0.001^**^	3.17 (1.70–5.95)	< 0.001^**^
Additional support^y^	0.43 (0.23–0.81)	0.009^*^		
Time since 1^st^ admission to the psychiatric hospital (in years)	0.99 (0.97–1.02)	0.746		

Odds ratio are presented with their 95% confidence interval (95% CI).

aOR, adjusted odds ratio, with the six variables included in the model.

^*^p < 0.05; ^**^p < 0.001; p-values are adjusted with the Holm method to limit multiple comparisons bias.

a No reference category is specified because the categories are not mutually exclusive. Results reflect the comparison of having the variable versus not. (Except for continuous variables in years with odds ratios reflecting a comparison of a 1-year difference).

y Occurrence within the year (12 months prior to hospitalization for cases or following index consultation for controls).

### 2.7 Data collection and ethical approval

Data were retrieved from the CHV’s electronic medical record system by reading through each medical record one by one. It was collected anonymously and entered directly into a secure document to ensure the confidentiality and privacy of participants. Personal identifying information such as names, addresses, and contact details were not recorded. Instead, each participant was assigned a unique identification code, which was used to perform the analyses with the studied variables. All data were stored securely and accessible only to authorized research personnel. Only the first author acquired data to guarantee reproducibility. Only the selected variables cited above were collected in the binary format “yes” or “no”, except for the two quantitative variables “age” and “time since first admission to the psychiatric hospital in outpatient or inpatient setting” collected in years (whole number).

To ensure data reliability, data were directly collected during the reading of each medical record.

Ethical approval was obtained by the Ethics Committee of the CHV with the registration number CEREVI/2023/003 on 27 February 2023. The study was conducted in accordance with the Declaration of Helsinki.

## 3 Results

### 3.1 Participants and missing data investigation

All eligible cases have been included in the study (216 cases). Based on the number of cases and the predetermined targeted sample size, 401 controls were included out of a total eligible population of 1,044. The included controls were randomly selected from the sample list of eligible controls.

No missing data were observed for *n* = 521 patients out of the 617 included in the study.

When considering the mechanism underlying these missing data, it is important to note that they predominantly pertain to variables that necessitate investigating past events. Specifically, these pertain to the presence or absence of a family history of mental health diseases (*n* = 55 missing data points out of 617, i.e., 8.9%), the presence or not of personal traumatic history (*n* = 38 missing data points out of 617, i.e., 6.2%), and whether or not there was a history of suicide attempt (*n* = 17 missing data out of 617, i.e., 2.8%). The other variables have less than 10 missing data points each. The details regarding missing data points for each of the variables within cases and controls are available in **Table 1**.

### 3.2 Sociodemographic characteristics and descriptive analysis

Data from *N* = 617 patients followed up in an outpatient setting from June 2021 to February 2023 have been investigated for descriptive analysis (216 cases and 401 controls). Men were a higher proportion of cases (65.3%) than controls (56.1%). Cases were slightly younger than controls, with a mean age of 42.7 years old versus 45.1 years, respectively. Unemployment was higher among cases than controls (75.9% of unemployment for cases versus 62.8% for controls), and in parallel, more people had unskilled work in the control group (18.0% versus 5.1% in the case group (*p* < 0.001)). Homelessness was much more prevalent among cases than controls, with 13.4% of homeless individuals among cases versus 2.5% for controls (*p* < 0.001).

There was a difference in proportion for the main psychiatric diagnosis between groups for depression, schizophrenia or other primary psychotic disorders, anxiety or fear-related disorders, and neurodevelopmental disorders. Schizophrenia, or other primary psychotic disorders, was the main diagnosed psychiatric disease in our population (66.7% and 51.4% for cases and controls, respectively, *p* < 0.001).

For historical issues and lifestyle variables: case and control groups significantly differed in proportion for history of mental healthcare without consent, multiple psychiatric hospitalization history (> 5), alcohol or illicit drug abuse within the year, family history of mental health disease, and history of attempted suicide (*p* < 0.001 except for family history of mental health disease with *p* = 0.007).

Finally, considering follow-up-related variables, strong significant proportion differences between groups for the following variables were observed (*p* < 0.001): visit to a psychiatric emergency, drug treatment discontinuation, medical follow-up discontinuation, and additional support (all within the year). For the variables: visit to psychiatric emergency, drug treatment discontinuation, and medical follow-up discontinuation, the rates were all higher among cases than controls with respectively 60.2%, 58.3%, and 49.1% (cases) versus 7.7%, 12.5%, and 14.0% (controls). Conversely, additional support had a higher proportion in controls (93.8%) than in cases (84.3%) (*p* < 0.001).

**Table 1** describes the detailed sociodemographic, clinical, personal history, and follow-up characteristics of cases and controls (*N* = 617).

### 3.3 Analytic statistics: multivariable modeling using multiple logistic regression

For the analytic statistics, modeling was conducted using a subset of patients without missing data (complete case analysis) with *n* = 521. According to our model, we found that six independent variables are significantly associated with full psychiatric hospitalization for patients being followed up in an outpatient setting. Indeed, in multivariable analysis, psychiatric hospitalization of outpatients remained strongly associated with a visit to a psychiatric emergency within a year (aOR: 13.02 [95% CI: 7.32–23.97]), a drug treatment or medical follow-up discontinuation within a year (aOR: 6.43 [95% CI: 3.52–12.03] and aOR: 3.17 [95% CI: 1.70–5.95], respectively), a history of mental healthcare without consent (aOR: 5.48 [95% CI: 3.10–10.06]), and a history of attempted suicide (aOR: 2.50 [95% CI: 1.48–4.30]). Finally, having a work (unskilled work) was conversely associated with a smaller risk of psychiatric hospitalization (aOR: 0.26 [95% CI: 0.10–0.65]). Estimates of adjusted odds ratio were calculated using logistic regression adjusted for the variables included in the model: “visit to a psychiatric emergency within a year”, “drug treatment discontinuation within a year”, “history of mental healthcare without consent”, “medical follow-up discontinuation within a year”, “history of attempted suicide”, and “unskilled job”.

**Table 2** presents these identified variables with their respective odds ratios and confidence intervals.

## 4 Discussion

This study aimed to identify and confirm variables associated with hospitalization, including both protective and risk factors. This information aims to guide and establish appropriate vigilance and follow-up care for mental health in an outpatient setting.

According to our multivariable logistic regression model, six variables have been independently found to be significantly associated with full hospitalization in psychiatry for patients followed up in an outpatient setting: visit to a psychiatric emergency within a year, drug treatment discontinuation within a year, history of mental healthcare without consent, medical follow-up discontinuation within a year, history of attempted suicide, and unskilled job. These findings highlight the importance of considering follow-up-related, historical issues and sociodemographic determinants for successful outpatient rehabilitation and, by extension, deinstitutionalization.

Visit to a psychiatric emergency within the year was the most strongly associated variable with hospitalization and had an aOR of 13.02 (95% CI: 7.32–23.97) in our model. This result is in line with literature that identified emergency visits associated with hospitalization, but to a lesser extent and not in an exclusive outpatient setting like in our study (21) (10). Drug treatment discontinuation within the year was associated with an aOR of 6.43 (95% CI: 3.52–12.03). A systematic literature review by Donisi et al. (11) identified medication compliance as a factor associated with readmissions of psychiatric patients, but Antonio Ciudad et al. (20) found conflicting results for schizophrenic outpatients. A recent study on early psychiatric rehospitalization also found mental health prescription adherence as a predictor of rehospitalization with a random forest analysis (10). Medication compliance is known to be an important and challenging factor in the care of psychiatric patients (22). Our study identified and confirmed the importance of medication compliance in an outpatient setting. History of mental healthcare without consent was also associated with hospitalization (aOR: 5.48, 95% CI: 3.10–10.06). We can assume that patients with a history of care without consent are the ones with bad insight into their illness and are therefore more complex patients, requiring more frequent hospitalization. This risk factor has already been identified, particularly in schizophrenic patients (23). In another study, conducted without distinction of psychiatric pathology and still in an inpatient setting, no statistical association was found (18). Medical follow-up discontinuation in psychiatry has also already been studied in the literature. Anne Nelson et al. examined whether patients discharged from inpatient psychiatric care (and not originated from outpatient care like in our study) would have lower rehospitalization rates if they kept an outpatient follow-up appointment after discharge (17). The authors showed a greater rate of rehospitalization for patients who did not keep an appointment after discharge. The same conclusions have been drawn on a general psychiatric inpatient population (10) and on a study focused on schizophrenia (14). In our study, where patients come from an outpatient setting, we also found that medical follow-up discontinuation is a risk factor for hospitalization (aOR: 3.17, 95% CI: 1.70–5.95). A history of attempted suicide also appeared to be a risk factor for psychiatric hospitalization for patients followed up in an outpatient mental health setting, with a 2.50 aOR (95% CI: 1.48–4.30). However, the literature shows conflicting results. Some studies also confirm this risk factor, which has previously been identified in studies conducted in inpatient settings (18, 24); in other studies, this risk factor was unclear, with nonsignificant results (11, 21, 25). The ability to have a job, which has been collected in our study with the variable “unskilled worker”, has been identified as a protective factor in the multivariable logistic regression model (*p*-value: 0.006) adjusted for potential confounders, as illustrated in **Table 2**: aOR of 0.26 (95% CI: 0.10–0.65). We explain this protective effect by assuming that controls, supposed to be clinically less severe than cases, with fewer symptoms, are more likely to get and keep a work. Having a job is indeed linked with cognitive remediation and the recovery process (26). “Unskilled worker” has been the only job category collected because other job categories were almost nonexistent in our population.

The community-based outpatient setting of the present study is particularly interesting regarding its population characteristics. Indeed, it offers multi-professional monitoring, which is valuable for patients with severe illnesses. With 75.9% of cases and 62.8% of controls unemployed in our study, this strongly suggests that mental disability significantly impacts psychosocial determinants, highlighting its importance. As with other chronic illnesses, psychological disability is a barrier to employment, and the severity of the condition is related to the ability to work (26). This might also explain the protective effect found in the association of the variable “unskilled worker”. Patients followed up regularly in this setting are also considered “severe” for other reasons. They often cannot follow a liberal mental health specialist due to poor socioeconomic conditions and may have a too severe psychiatric disorder requiring hospital practitioners (due to complex pharmacotherapeutics or illness) to reach a stable medical state. From a clinical point of view, most patients having a main diagnosis of schizophrenia or other primary psychotic disorders (66.7% among cases and 51.4% among controls) is another argument for the population severity, with patients who cannot be adequately followed up by general practitioners and/or private psychiatrists. Interestingly, this does not represent the psychiatric diseases distribution of general population and is even the opposite. Indeed, in France, anxiety disorders have the highest prevalence, followed by depression, bipolar disorders, and finally, psychotic disorders (27). Regarding historical issues and lifestyle, the prevalence of traumatic history was notably high in both groups, with around 60% prevalence. Mental health conditions are well-known to have multifactorial origins (28). Nevertheless, it is noteworthy to observe the prevalence of traumatic exposure within our study population. The high proportions of patients with mental healthcare without consent history and multiple psychiatric hospitalization histories (> 5) also underline the specificities of our outpatient population, which have a certain severity. Multidisciplinary community-based care has the potential to address the specific needs of the population within the framework of deinstitutionalization when considering the identified determinants.

The case–control design and the multivariable logistic regression utilized have, however, their limitations. Firstly, the population selection has been made through “hospital recruitment” (outpatient service attached to the CHV public psychiatric hospital). It can therefore introduce a selection bias regarding the admission probability of participants to that public outpatient service (e.g., patients with poorest socioeconomic conditions). Nonetheless, as the probability of admission to that service relies on the geographical sectorization (population originating from a defined geographic urban area: third, sixth, and eighth districts of Lyon) and has few equivalents in the private sector, we consider this bias to be existent but limited. To limit classification bias, classification was made on electronical medical records identically for cases and controls. Sectorization also prevents the risk of missing a hospitalization in another facility by ensuring the patient is ultimately hospitalized in his or her local hospital. Confusion bias has been considered via modeling with multivariable logistic regression. We assessed interactions in our model with one being significant (variable history of mental healthcare without consent with variable history of attempted suicide, adjusted *p*-value of 0.004). We, however, decided not to include this interaction in the model because (i) the clinical relevance of this interaction was not key in our exploratory investigation, and we do not seek a predictive model; (ii) considering that this interaction barely improves our overall model significance (residual deviance of 361 when considered versus 370, *p*-value: 0.003). Lastly, a limitation of our model is the absence of residuals normality for this multiple logistic regression. Indeed, residuals do not seem independent of the predicted values. Some explanatory variables would thus be lacking and not exhaustively listed in this study, such as variables on education level or on patient’s attitude and perception.

The highlights of this study are, however, its overall consistency with literature data on previously identified risk factors associated with hospitalization and the confirmation of these factors in an exclusive outpatient setting. The recruitment method used in this study with the sectorization principle of the service is also a robust point because it allowed to limit selection bias and consider all the patients followed up in this special outpatient setting.

## 5 Conclusion

Our study identified several independent risk and protective factors for hospitalization among patients with a mental health condition who are being treated in an outpatient setting. These factors include variables related to follow-up, such as a recent visit to a psychiatric emergency and recent discontinuation of drug treatment or medical follow-up (within the year), as well as historical issues or lifestyle-related factors.

To our knowledge, this is the first time that these factors are assessed statistically together in a specific outpatient setting, with patients not originating exclusively from a hospital. That is of great interest in the deinstitutionalization era. Public health policies at local and to a bigger extent, at the national scale, should consider these new data to target and tailor appropriate follow-up of care in outpatient settings. Tools to distinguish patients with the identified risk factors and prevent them from being hospitalized should also be created and adapted.

## Data availability statement

The data analyzed in this study is subject to the following licenses/restrictions: Medical information that cannot be shared according to the ethical approval obtained by the Ethics Committee of the CHV with the registration number CEREVI/2023/003 on 02/27/2023. Requests to access these datasets should be directed to matthieu.lebrat@hotmail.fr.

## Ethics statement

The studies involving humans were approved by Ethics Committee of the CHV with the registration number CEREVI/2023/003 on 02/27/2023. The studies were conducted in accordance with the local legislation and institutional requirements. The ethics committee/institutional review board waived the requirement of written informed consent for participation from the participants or the participants’ legal guardians/next of kin because the data were obtained in routine care practice with patient information and possible retraction. The study was carried out in accordance with current legislations.

## Author contributions

ML: Writing – review & editing, Writing – original draft, Visualization, Validation, Software, Resources, Project administration, Methodology, Investigation, Formal Analysis, Data curation, Conceptualization. RM: Writing – review & editing, Supervision, Resources, Project administration, Funding acquisition, Conceptualization. CD: Writing – review & editing, Visualization, Validation, Supervision, Methodology. LZ: Writing – review & editing, Validation, Supervision, Project administration. JP: Writing – review & editing, Software, Data curation. NF: Writing – review & editing, Validation, Supervision, Resources, Project administration, Funding acquisition.
